# Alternate-Day Ketogenic Diet Feeding Protects against Heart Failure through Preservation of Ketogenesis in the Liver

**DOI:** 10.1155/2022/4253651

**Published:** 2022-06-06

**Authors:** Yanjie Guo, Xiaoxie Liu, Tao Li, Juanhua Zhao, Yanni Yang, Yanni Yao, Lan Wang, Beibei Yang, Gui Ren, Yanzhen Tan, Shan Jiang

**Affiliations:** ^1^Department of Cardiology, Xi'an International Medical Center Hospital, Xi'an 710100, China; ^2^Department of Rehabilitation Medicine, Peking University Third Hospital, Beijing 100191, China; ^3^Ultrasound Diagnostic and Treatment Center, Digestive Disease Hospital, Xijing Hospital, Fourth Military Medical University, Xi'an 710032, China; ^4^National Clinical Research Center for Digestive Diseases and Xijing Hospital of Digestive Diseases, Fourth Military Medical University, Xi'an 710032, China; ^5^Department of Cardiovascular Surgery, Xijing Hospital, Fourth Military Medical University, Xi'an 710032, China; ^6^Department of Rehabilitation Medicine, China-Japan Friendship Hospital, Beijing 100029, China

## Abstract

As heart failure develops, the heart utilizes ketone bodies at increased rates, indicating an adaptive stress response. Thus, increasing ketone body availability exerts protective effects against heart failure. However, although it is the widely used approach for increasing ketone body availability, the ketogenic diet shows limited cardioprotective effects against heart failure. This study was aimed at examining the effects of the ketogenic diet on heart failure and the underlying mechanisms. Pressure overload-induced heart failure was established by transverse aortic constriction (TAC) in mice. Continuous ketogenic diet feeding for 8 weeks failed to protect the heart against heart failure. It showed no significant effects on cardiac systolic function and fibrosis but aggravated cardiac diastolic function in TAC mice. Specifically, it induced systemic lipid metabolic disorder and hepatic dysfunction in TAC mice. It decreased the content of 3-hydroxy-3-methylglutaryl-CoA lyase (HMGCL), a key enzyme in ketogenesis, and impaired the capacity of hepatic ketogenesis in TAC mice. It preserved the capacity of hepatic ketogenesis and exerted cardioprotective effects against heart failure, increasing cardiac function and decreasing cardiac fibrosis, in liver-specific HMGCL-overexpressed TAC mice. Importantly, we found that alternate-day ketogenic diet feeding did not impair the capacity of hepatic ketogenesis and exerted potent cardioprotective effects against heart failure. These results suggested that alternate-day but not continuous ketogenic diet protects against heart failure through preservation of ketogenesis in the liver.

## 1. Introduction

The heart has developed a strong metabolic system capable of utilizing almost all substrates, including fatty acids, carbohydrates, lactate, ketone bodies, and amino acids, to sustain high energy demands. In the fasting state, the heart primarily relies on fatty acid oxidation (~60-90%) and shifts its preference to other substrates in response to different conditions [1]. For example, it shifts its preference to a greater reliance on glucose with a concomitant suppression of fatty acid utilization in the failing heart [2]. Derangements in cardiac substrate utilization play a causal role in the development of heart failure, and reprogramming cardiac substrate preference is a promising strategy for the treatment of cardiac disease [3–6].

Recent studies have shown that the utilization of ketone bodies is increased in the failing heart in both rodents and humans [7–9]. Infusion of the ketone body *β*-hydroxybutyrate (*β*-OHB) improved cardiac function in the failing heart [10–12]. Additionally, acute infusion of *β*-OHB exerts impressive hemodynamic improvement in humans with heart failure [13]. These advances suggest that enhancement of cardiac ketone body utilization is a potential strategy for the treatment of heart failure. Ketogenic diet (KD) feeding, the most widely used approach to increase circulating levels of ketone bodies, has been extensively studied in recent years. KD feeding exerts extensive beneficial effects, including lifespan extension, glucose tolerance improvement, and cardiac protection [14, 15]. However, chronic KD feeding imbalances fatty acid metabolism, resulting in adverse effects [15]. We noticed that several studies reported that chronic KD feeding failed to protect the heart against heart failure in rodents. Feeding mice with a KD for 4 weeks only ameliorated pathological cardiac remodeling without improving cardiac function during heart failure, but infusion of 3-hydroxybuytrate or ketone ester showed significant protective effects [10, 11]. Additionally, the results of clinical studies designed to test the benefits of KD for cardiac disease or cardiovascular disease risk factors are still inconclusive [16–19]. The underlying mechanism of a poor cardioprotective effect of KD feeding during heart failure remains unknown.

The study was aimed at examining the effects of KD feeding on cardiac function in mice with heart failure and its underlying mechanism, and we found that continuous KD feeding decreased the capacity of hepatic ketogenesis and failed to improve cardiac function in mice with heart failure. Importantly, we found that alternate-day KD feeding (feeding the mice with KD every other day) preserved the hepatic ketogenesis capacity and exerted cardioprotective effects against heart failure. These findings suggested that alternate-day but not continuous KD feeding is a potential strategy for heart failure treatment.

## 2. Methods

### 2.1. Animals and Heart Failure Model

All animal experiments conformed to the national guidelines and were approved by the Xi'an International Medical Center Hospital Animal Care and Use Committee. Transverse aortic constriction (TAC) surgery was used to establish pressure overload-induced heart failure in C57BL/6J mice (male, 8 weeks old) as described previously [20]. Briefly, mice were anesthetized with inhaled isoflurane (2% in 100% oxygen), and the aorta was occluded using a 7-0 ligature. We used a 27-gage needle to ensure consistent occlusion. Sham mice underwent the same surgery but without occlusion. After surgery, mice were fed with a normal diet (ND, consisting of 10%, 10%, and 80% calories from protein, fat, and carbohydrates, respectively) or KD (consisting of 10%, 90%, and 0% calories from protein, fat, and carbohydrates, respectively) for 2 months. Both ND (D10070802) and KD (D10070801) were obtained from Research Diets Inc. (NJ, USA). For continuous KD feeding, mice were fed with KD consistently for 2 months. For alternate-day KD feeding, mice were fed with KD every other day (KD for one day, and ND for the other day). Cardiac function and other parameters were detected at 8 weeks postsurgery.

### 2.2. Adeno-Associated Virus (AAV) Infection

AAV8 encoding 3-hydroxy-3-methylglutaryl-CoA lyase (HMGCL) was provided by Hanheng Biotechnology (Shanghai, China). Mice (male, 4 weeks old) were randomly chosen to receive a single-bolus tail vein injection of either AAV8 encoding HMGCL or AAV8 encoding GFP at 1 × 10^11^ viral genomes per mouse. After 4 weeks, the mice were subjected to sham or TAC surgery.

### 2.3. Determination of Cardiac Function

Transthoracic echocardiography was performed using a VisualSonics Vevo 2100 high-resolution in vivo imaging system (VisualSonics Inc., Toronto, Canada) as described previously [21]. Mice were anesthetized by isoflurane (1%) with maintenance of stable heart rate and body temperature. The heart rate was maintained at 415–460 beats per min. LV systolic function and other indices of systolic function were determined from short-axis M-mode scans at the midventricular level, as indicated by the presence of papillary muscles. Apical four-chamber views were obtained for diastolic function measurements using pulsed-wave and tissue Doppler imaging at the mitral valve level. The ratio between early mitral inflow velocity and mitral annular early diastolic velocity (*E*/*e*′) was used to assess cardiac diastolic function.

### 2.4. Cell Cultures and Treatments

HepG2 cells were used for cellular experiments. Cells were cultured in Dulbecco's Modified Eagle's Medium (Invitrogen) containing 10% fetal bovine serum (BioInd) and 1% penicillin/streptomycin solution (Hyclone) under 5% CO_2_ at 37°C. HepG2 cells were treated with palmitic acid (PA, 500 *μ*M) or oleic acid (OA, 500 *μ*M) for 24 h unless otherwise indicated. For mitochondrial ROS scavenger treatment, HepG2 cells were treated with MitoTEMPO (1 *μ*M) or SS31 (50 *μ*M) for 24 h. Adenovirus-encoding HMGCL was used to overexpress HMGCL in HepG2 cells. After 60 h of infection, cells were treated with PA or OA for 24 h. Mitochondrial ROS was detected using a mitochondrial ROS assay kit (Cayman) following the manufacturer's instructions.

### 2.5. Biochemical Assays

*β*-OHB contents in plasma and the liver were measured with a *β*-hydroxybutyrate assay kit (Sigma-Aldrich, MAK041) according to the manufacturer's instructions. Total cholesterol (TCHO), triglyceride (TG), and nonesterified free fatty acid (FFA) concentrations in tissues and plasma were quantified using commercial assay kits (Nanjing Jiancheng, China) according to the manufacturer's instructions. Plasma and liver alanine aminotransferase (ALT) and aspartate aminotransferase (AST) were measured with the ALT Assay Kit and AST Assay Kit (Nanjing Jiancheng, China), respectively, according to the manufacturer's instructions. Cardiac ROS levels were measured with the OxiSelect in vitro ROS Assay Kit (Cell Biolabs) according to the manufacturer's instructions.

### 2.6. Histological Analysis

Cardiac and hepatic tissues were collected after anesthesia of mice with sodium pentobarbital (400 mg/kg, i.p.) and then fixed in paraformaldehyde solution (4%). Tissues were paraffin embedded and sectioned at a thickness of 5 *μ*m. Masson staining and wheat germ agglutinin (WGA) staining were performed to assess cardiac fibrosis and hypertrophy, respectively, as described previously [6]. Haematoxylin-eosin (H&E) and Oil Red staining were performed to assess structural changes and lipid accumulation in the liver, respectively, as described previously [22]. The fluorescence of WGA was imaged using an inverted confocal microscope (Zeiss LSM 800).

### 2.7. Assessment of Voluntary Physical Activity

Voluntary physical activity was assessed by voluntary wheel running. One day before assessment, mice were habituated in polycarbonate cages with free access to stainless steel wheels (diameter 11.75 cm). The wheels were wirelessly connected to a hub in the computer with automatic recording of the distance ran by each mouse. The data recorded during the 2 succeeding days were analyzed.

### 2.8. Western Blot

Protein expression was measured using Western blot as described previously [23]. The immunoblots were probed with anti-CD36, anticarnitine palmitoyltransferase 1b (CPT1b), anti-HMGCL, anti-3-hydroxy-3-methylglutaryl-CoA synthase 2 (HMGCS2), anti-3-hydroxybutyrate dehydrogenase 1 (BDH1), anticatalase, antiglutathione peroxidase 1 (GPX1), antisuperoxide dismutase 2, mitochondrial (SOD2), or anti-*β*-actin (Cell Signaling Technology) overnight at 4°C followed by incubation with the corresponding secondary antibodies at room temperature for 1 h. The blots were visualized with ECL Plus reagent.

### 2.9. Statistical Analysis

All values are presented as mean ± SEM. The significance of differences was determined by an unpaired, two-tailed Student's *t*-test or a two-way analysis of variance followed by a Bonferroni post hoc analysis where appropriate. Differences were considered significant when *P* < 0.05.

## 3. Results

### 3.1. Continuous KD Feeding Failed to Improve Cardiac Function in TAC Mice

**Figure 1:**
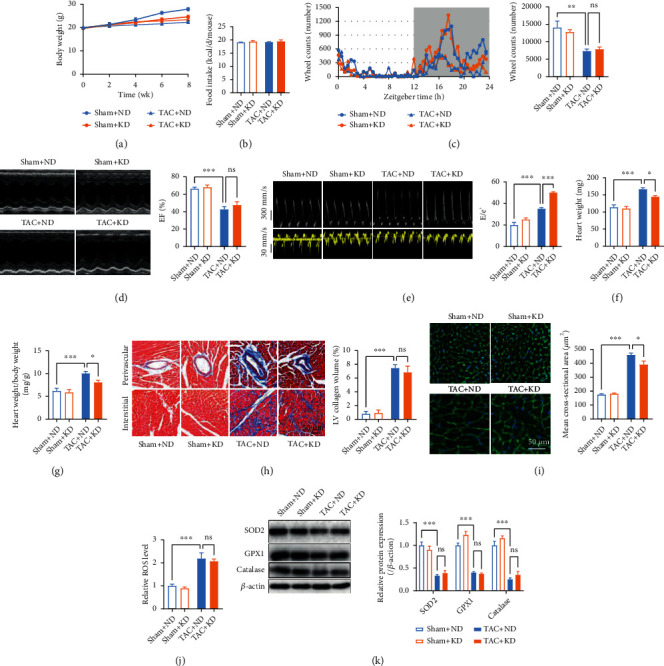
Continuous KD feeding failed to protect the heart against heart failure. (a) Body weight of sham and TAC mice postsurgery. (b) Food intake of sham and TAC mice. (c) Voluntary physical activity as assessed by voluntary wheel running in sham and TAC mice. (d) Echocardiography revealed that KD feeding showed no significant effects on LV systolic function in TAC mice. (e) Representative pulsed-wave Doppler (top) and tissue Doppler (bottom) tracings. The ratio between early mitral inflow velocity and mitral annular early diastolic velocity (*E*/*e*′) was quantified in the right. (g) Heart weight/body weight of sham and TAC mice. (h) KD feeding failed to attenuate cardiac fibrosis in TAC mice. (i) KD feeding decreased the cross-sectional area of cardiomyocytes in TAC mice. (j) Cardiac ROS levels in sham and TAC mice. (k) Cardiac expressions of SOD2, GPX1, and catalase in sham and TAC mice. (b–k) Experiments were performed at 8 weeks postsurgery. *n* = 8. ^∗^*P* < 0.05; ^∗∗^*P* < 0.01; ^∗∗∗^*P* < 0.0001.

Sham and TAC mice were fed with either ND or KD for 8 weeks postsurgery to test whether KD feeding protects the heart against heart failure. TAC mice displayed lower body weight and lesser voluntary physical activity than age-matched sham mice (Figures 1(a)–1(c)). KD feeding showed no significant effects on body weight, food intake, and voluntary physical activity in TAC mice (Figures 1(a)–1(c)). Pressure overload decreased cardiac function as evidenced by decreased LV systolic function and impaired LV diastolic function (Figures 1(d) and 1(e)). Although KD feeding showed no significant effects on LV systolic function, it aggravated LV diastolic dysfunction in TAC mice (Figures 1(d) and 1(e)). In addition, pressure overload induced cardiac hypertrophy as evidenced by increased heart weight, heart weight/body weight, cardiac fibrosis, and cross-sectional area of cardiomyocytes (Figures 1(f)–1(i)). Although KD feeding decreased cardiac hypertrophy as evidenced by decreased heart weight, heart weight/body weight, and cross-sectional area of cardiomyocytes, it failed to attenuate cardiac fibrosis in TAC mice (Figures 1(f)–1(i)). Furthermore, pressure overload increased cardiac ROS level and decreased cardiac antioxidant enzymes including SOD2, GPX1, and catalase, which were not improved by KD feeding (Figures 1(j) and 1(k)). These results suggested that continuous KD feeding failed to protect the heart against heart failure, and it aggravated cardiac diastolic function in TAC mice.

### 3.2. Continuous KD Feeding Decreased the Capacity of Hepatic Ketogenesis in TAC Mice

**Figure 2:**
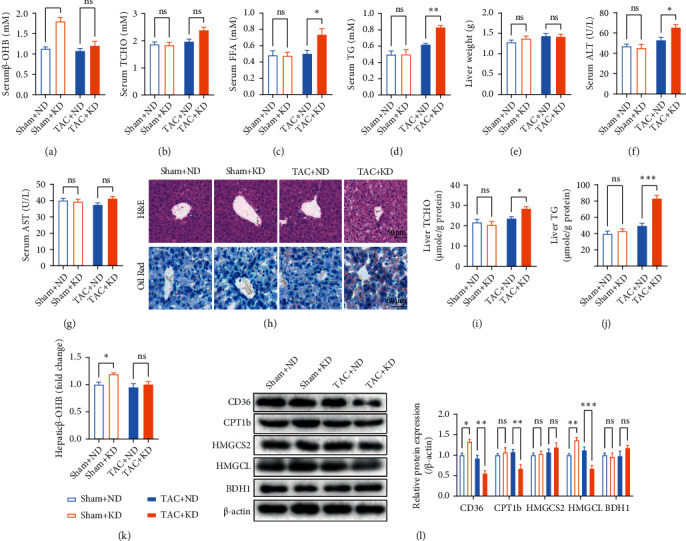
Continuous KD feeding impaired the capacity of hepatic ketogenesis in TAC mice. (a) Serum *β*-OHB concentrations in sham and TAC mice. (b–d) Circulating total cholesterol (TCHO) (b), free fatty acid (FFA) (c), and triglyceride (TG) (d) in sham and TAC mice. (e) Liver weight in sham and TAC mice. (f) Serum alanine aminotransferase (ALT) (f) and aspartate aminotransferase (AST) (g) in sham and TAC mice. (h) H&E and Oil Red staining in liver sections from sham and TAC mice. (i, j) TCHO (i) and TG (j) contents from the liver of sham and TAC mice. (k) KD feeding increased the hepatic *β*-OHB content in sham mice but not in TAC mice. (l) Contents of CD36, CPT1b, HMGCS2, HMGCL, and BDH1 from the liver of sham and TAC mice. All experiments were conducted at 8 weeks postsurgery. *n* = 6. ^∗^*P* < 0.05; ^∗∗^*P* < 0.01; ^∗∗∗^*P* < 0.0001.

KD feeding increased serum *β*-OHB concentrations in sham mice but showed no significant effect in TAC mice (Figure 2(a)), indicating that continuous KD feeding failed to increase circulating ketone bodies during heart failure. Detection of circulating lipids showed that KD feeding showed no significant effects on TCHO, FFA, and TG in sham mice, but it increased these lipids in TAC mice (Figures 2(b)–2(d)). Considering that liver plays a critical role in lipid metabolism regulation, hepatic function was evaluated. KD feeding showed no significant effects on liver weight and serum AST in both sham and TAC mice, but it increased serum ALT in TAC mice (Figures 2(e)–2(g)), suggesting hepatic dysfunction in KD-fed TAC mice. This result was further validated by H&E and Oil Red staining, which showed apparent steatosis and lipid deposition from the liver of KD-fed TAC mice (Figure 2(h)). In addition, KD feeding increased the TCHO and TG contents in the liver of TAC mice (Figures 2(i) and 2(j)). Consequently, KD feeding increased the hepatic *β*-OHB content in sham mice but not in TAC mice (Figure 2(k)). Detection of fatty acid metabolism- and ketogenesis-related proteins in the liver showed that KD feeding increased CD36 (a key protein in regulating fatty acid uptake) and HMGCL (a key enzyme in ketogenesis) contents in sham mice, while it decreased CD36, CPT1b (a protein facilitating fatty acid translocation across the mitochondrial membrane), and HMGCL contents in TAC mice (Figure 2(l)). These results suggested that KD feeding impaired the hepatic ketogenesis capacity during heart failure.

### 3.3. Chronic Fatty Acid Treatment Decreased HMGCL Content and Ketogenesis in Cultured Hepatocytes

**Figure 3:**
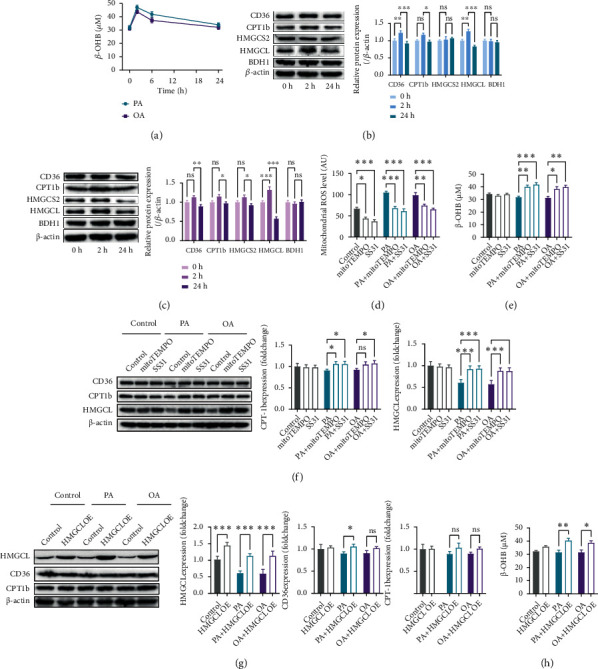
Chronic fatty acid treatment decreased HMGCL content and ketogenesis in cultured hepatocytes. (a) *β*-OHB content in the culture medium at 2, 6, and 24 h post-PA and -OA treatments. (b) CD36, CPT1b, HMGCS2, HMGCL, and BDH1 contents at 2 and 24 h post-PA treatment. (c) CD36, CPT1b, HMGCS2, HMGCL, and BDH1 contents at 2 and 24 h post-OA treatment. (d) Mitochondrion-targeted antioxidants, MitoTEMPO and SS31, decreased mitochondrial ROS in untreated, PA-treated, and OA-treated hepatocytes. (e) Scavenging mitochondrial ROS using MitoTEMPO and SS31 increased *β*-OHB content in the culture medium at 24 h post-PA and -OA treatments. (f) Scavenging mitochondrial ROS increased the HMGCL content in cultured hepatocytes at 24 h post-PA and -OA treatments. (g) HMGCL overexpression showed no significant effects on the CD36 and CPT1b contents in hepatocytes at 24 h post-PA and -OA treatments. (h) HMGCL overexpression increased *β*-OHB content in the culture medium at 24 h post-PA and -OA treatments. *n* = 6. ^∗^*P* < 0.05; ^∗∗^*P* < 0.01; ^∗∗∗^*P* < 0.0001.

To test whether continuous fatty acid flux decreases the capacity of hepatic ketogenesis, the effects of chronic fatty acid treatment on ketogenesis in cultured HepG2 cells were detected. The cultured hepatocytes were treated with PA or OA for 2, 6, and 24 h. Both PA and OA increased *β*-OHB content in the culture medium at 2 h posttreatment, and this effect was reversed at 24 h posttreatment (Figure 3(a)). Regarding the key proteins in fatty acid metabolism and ketogenesis, PA increased the contents of CD36, CPT1b, and HMGCL at 2 h posttreatment and decreased the HMGCL content at 24 h posttreatment in cultured hepatocytes (Figure 3(b)). Similarly, OA increased the HMGCL content at 2 h posttreatment and decreased the HMGCL content at 24 h posttreatment in cultured hepatocytes (Figure 3(c)). Given that upregulation of mitochondrial ROS plays a critical role in the induction of metabolic disorder in the liver [24, 25], we examined whether mitochondrial ROS is involved in chronic fatty acid treatment-impaired ketogenesis in hepatocytes. Both PA and OA increased the mitochondrial ROS, and mitochondrion-targeted antioxidants, namely, MitoTEMPO and SS31, decreased mitochondrial ROS in untreated, PA-treated, and OA-treated cells (Figure 3(d)). MitoTEMPO, a small compound, is reported to scavenge mitochondrial superoxide specifically, while SS31, a mitochondrion-targeted peptide, is reported to scavenge ROS in general and H_2_O_2_ and ONOO^−^ specifically [26]. Importantly, scavenging mitochondrial ROS using MitoTEMPO or SS31 increased the *β*-OHB content in the culture medium and increased the HMGCL content in cultured hepatocytes at 24 h post-PA and -OA treatments (Figures 3(e) and 3(f)), indicating an important role of HMGCL in chronic PA and OA treatment-induced impairment in ketogenesis. Thus, HMGCL was overexpressed using adenovirus in cultured hepatocytes. HMGCL overexpression showed no significant effects on CD36 and CPT1b contents in hepatocytes but increased the *β*-OHB content in the culture medium at 24 h post-PA and -OA treatments (Figures 3(g) and 3(h)). These results suggested that chronic fatty acid flux decreased the BDH1 expression and ketogenesis in hepatocytes.

### 3.4. Liver-Specific Overexpression of HMGCL Restored the Cardioprotective Effects of KD Feeding in TAC Mice

**Figure 4:**
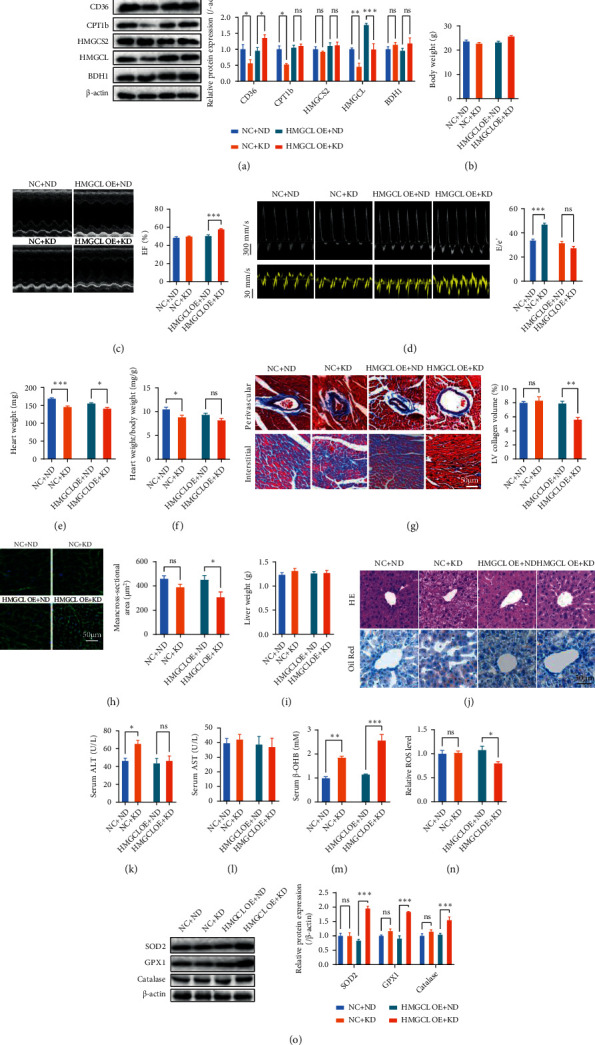
Liver-specific overexpression of HMGCL restored the cardioprotective effects of KD feeding in TAC mice. (a) CD36, CPT1b, HMGCS2, HMGCL, and BDH1 contents in WT and liver-specific HMGCL-overexpressed TAC mice. (b) Body weight of WT and liver-specific HMGCL-overexpressed TAC mice. (c, d) KD feeding increased cardiac systolic function (c) without affecting cardiac diastolic function (d) in liver-specific HMGCL-overexpressed TAC mice. (e–h) KD feeding decreased heart weight (e), heart weight/body weight (f), cardiac fibrosis (g), and the cross-sectional area of cardiomyocytes (h) in liver-specific HMGCL-overexpressed TAC mice. (i) Liver weight of WT and liver-specific HMGCL-overexpressed TAC mice. (j) H&E and Oil Red staining in liver sections from WT and liver-specific HMGCL-overexpressed TAC mice. (k, l) Serum ALT (k) and AST (l) in WT and liver-specific HMGCL-overexpressed TAC mice. (m) Circulating *β*-OHB levels in WT and liver-specific HMGCL-overexpressed TAC mice. (n) Cardiac ROS levels in WT and liver-specific HMGCL-overexpressed TAC mice. (o) Cardiac expressions of SOD2, GPX1, and catalase in WT and liver-specific HMGCL-overexpressed TAC mice. *n* = 8. ^∗^*P* < 0.05; ^∗∗^*P* < 0.01; ^∗∗∗^*P* < 0.0001.

Next, we assessed whether the liver-specific overexpression of HMGCL restores the cardioprotective effects of KD feeding in TAC mice. HMGCL was liver-specific overexpressed using AAV8 (Figure 4(a)). HMGCL overexpression showed no significant effects on body weight and cardiac function in ND-fed TAC mice (Figures 4(b)–4(h)). Although KD feeding failed to protect the heart against heart failure in wild-type (WT) mice, it exerted cardioprotective effects against heart failure in liver-specific HMGCL-overexpressed mice (Figures 4(c)–4(h)). KD feeding increased cardiac systolic function without affecting the cardiac diastolic function in liver-specific HMGCL-overexpressed TAC mice (Figures 4(c) and 4(d)). It decreased heart weight, heart weight/body weight, cardiac fibrosis, and the cross-sectional area of cardiomyocytes in liver-specific HMGCL-overexpressed TAC mice (Figures 4(e)–4(h)). Detection of hepatic function revealed that KD feeding showed no significant effects on liver weight and did not increase steatosis and lipid deposition in livers from HMGCL-overexpressed TAC mice (Figures 4(i) and 4(j)). KD feeding also showed no significant effects on serum ALT and AST in liver-specific HMGCL-overexpressed TAC mice (Figures 4(k) and 4(l)). In addition, KD feeding increased circulating *β*-OHB levels, decreased cardiac ROS levels, and increased cardiac antioxidant enzymes in HMGCL-overexpressed TAC mice (Figures 4(m)–4(o)). These results suggested that the liver-specific overexpression of HMGCL attenuated hepatic dysfunction and restored the cardioprotective effects of KD feeding in TAC mice.

### 3.5. Alternate-Day KD Feeding Protected the Heart against Heart Failure

**Figure 5:**
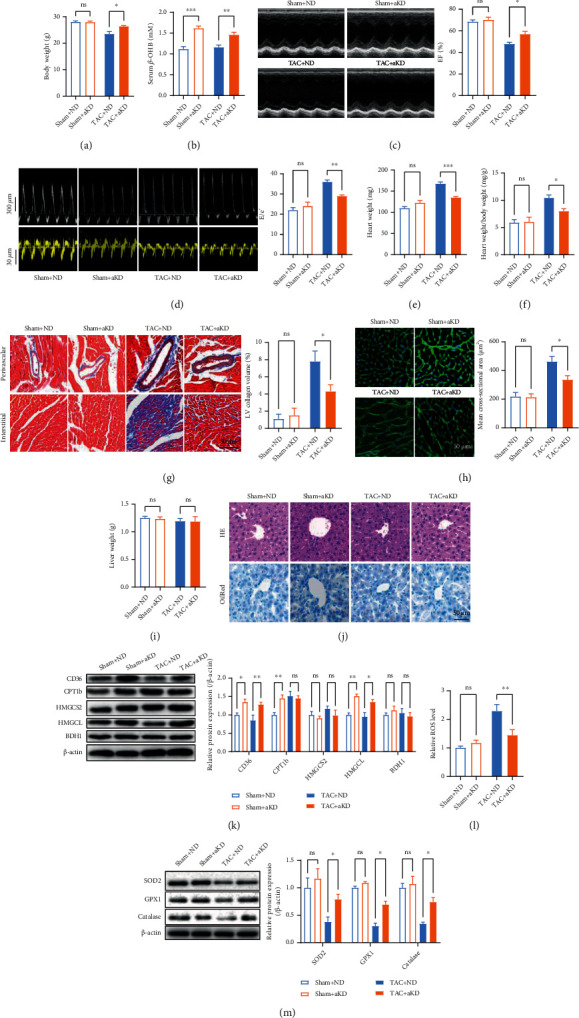
Alternate-day KD feeding protected the heart against heart failure. (a) Alternate-day KD feeding restored body weight in TAC mice but showed no significant effects in sham mice. (b–d) Alternate-day KD feeding increased *β*-OHB content in both sham and TAC mice (b), cardiac systolic function in TAC mice (c), and cardiac diastolic function in TAC mice (d). (e–h) Alternate-day KD feeding decreased heart weight (e), heart weight/body weight (f), cardiac fibrosis (g), and the cross-sectional area of cardiomyocytes (h) in TAC mice. (i, j) Alternate-day KD feeding showed no significant effects on weight (i), steatosis, and lipid deposition (j) in the liver of TAC mice. (k) Alternate-day KD feeding increased CD36 and HMGCL content in the liver of TAC mice. (l) Cardiac ROS levels in sham and TAC mice. (m) Cardiac expressions of SOD2, GPX1, and catalase in sham and TAC mice. *n* = 8. ^∗^*P* < 0.05; ^∗∗^*P* < 0.01; ^∗∗∗^*P* < 0.0001.

Given that continuous KD feeding failed to protect the heart against heart failure for hepatic dysfunction, we wonder whether KD feeding has an alternative way to preserve the hepatic function. Hence, an alternate-day KD feeding was employed. Results showed that alternate-day KD feeding restored body weight in TAC mice but showed no significant effects in sham mice (Figure 5(a)). Importantly, alternate-day KD feeding increased the *β*-OHB content in both sham and TAC mice (Figure 5(b)), suggesting that ketogenesis capacity was preserved. It also improved cardiac systolic function, as well as diastolic function (Figures 5(c) and 5(d)), and decreased heart weight, heart weight/body weight, cardiac fibrosis, and the cross-sectional area of cardiomyocytes in TAC mice (Figures 5(e)–5(h)). These results suggested that alternate-day KD feeding exerted potent cardioprotective effects against heart failure. Furthermore, alternate-day KD feeding showed no significant effects on weight, steatosis, and lipid deposition in the liver of TAC mice (Figures 5(i) and 5(j)). Alternate-day KD feeding increased CD36 and HMGCL contents in the liver of TAC mice (Figure 5(k)). It also decreased cardiac ROS levels and increased the expressions of cardiac antioxidant enzymes in TAC mice (Figures 5(l) and 5(m)). Thus, these results suggested that alternate-day KD feeding preserved the hepatic function and exerted cardioprotective effects in TAC mice.

## 4. Discussion

Although KD feeding has been widely reported to have numerous beneficial effects, undesirable effects such as aggravating metabolic disorder in some pathological conditions have also been observed [15]. The present study found that continuous KD feeding failed to protect the heart against heart failure, consistent with previous studies [10, 11], because it impaired the capacity of hepatic ketogenesis. Conversely, alternate-day KD feeding exerted cardioprotective effects against heart failure by preserving the capacity of hepatic ketogenesis. These results suggested that continuous KD feeding should be used with caution in the treatment of heart failure and alternate-day KD feeding is an alternative strategy for heart failure treatment.

During heart failure development, the heart utilizes ketone bodies at increased rates [7, 8], compensating for the reduced capacity of fatty acid utilization. Increased utilization of ketone bodies is reportedly an adaptive rather than maladaptive stress response during heart failure, reducing peripheral resistance and exerting anti-inflammatory and antioxidative effects [10, 11, 27–31]. Therefore, inhibition of cardiac ketone body utilization aggravates cardiac dysfunction, and increasing ketone body availability exerts cardioprotective effects during heart failure. However, as the most widely used approach for increasing ketone body availability, the cardioprotective effects of KD feeding against heart failure are inconsistent. We noticed that several studies reported that chronic KD feeding failed to protect the heart against heart failure [10, 11], consistent with our findings, suggesting that chronic KD feeding is not a reliable strategy for cardioprotection during heart failure. The potential adverse effects of chronic KD feeding have been widely reported, including decreased appetite, fatigue, weakness, gastrointestinal disturbances, cardiac arrhythmias, nephrolithiasis, constipation, muscle cramps, headaches, bone fracture, and vitamin deficiency [32–35]. These adverse effects of chronic KD feeding may be associated with the fatty acid metabolic disorder. The current study found that continuous KD feeding increased the levels of circulating lipids, as well as hepatic lipid deposition, in TAC mice, suggesting systemic fatty acid metabolic disorder. Fatty acid metabolic disorder is a risk factor of various diseases, including cardiovascular disease [36–38]. Thus, the capacity of the body to maintain lipid homeostasis is important in the biological effects of KD feeding.

We found that the failure of KD feeding in the protection of the heart against heart failure is due to the impairment of hepatic ketogenesis in TAC mice. Continuous KD feeding failed to increase circulating ketone bodies in mice with heart failure, as a result of dysfunction in lipid metabolism and ketogenesis in the liver. The liver is a major source of ketone bodies through ketogenesis in response to fatty acid influx [39]. After fatty acids have been catabolized into acetyl-CoA, acetyl-CoA is converted into acetoacetyl-CoA by *β*-ketothiolase. Subsequently, HMGCS2 converts acetoacetyl-CoA into *β*-hydroxy-*β*-methylglutaryl-CoA, which is eventually converted into acetoacetate by HMGCL. Acetoacetate can be reduced into *β*-OHB in a reversible manner by BDH1 [40, 41]. After hepatic ketogenesis, ketone bodies are released from the liver into the circulation and utilized by extrahepatic tissues through ketolysis. Here, we showed that continuous KD feeding increased the expression of key molecules in fatty acid metabolism and ketogenesis in sham mice, thereby increasing the circulating *β*-OHB, while these proteins were reduced in TAC mice. In particular, HMGCL deficiency contributed to the impairment in hepatic ketogenesis in KD-fed TAC mice. Continuous KD feeding decreased the HMGCL content in the liver of TAC mice, possibly because of mitochondrial oxidative stress. HMGCL overexpression preserved the capacity of ketogenesis and exerted cardioprotective effects in continuous KD-fed mice with heart failure. Although the role of HMGCL during heart failure and other pathological conditions is still poorly studied [42, 43], the results of the present study suggested that hepatic ketogenesis is impaired by continuous KD feeding and that the ketogenesis impairment is responsible for the adverse effects of KD feeding during heart failure. It should be noted that a ketonic diet induces complex responses, and other responses include decreasing insulin levels and increasing cortisol and glucagon levels, thus also disturbing glucose metabolism [44]. It is reported that glucose metabolic disorder also contributes to the development of heart failure [45]. Whether these changes induced by KD feeding are involved in the adverse effects of KD feeding during heart failure needs to be further studied.

Considering that continuous KD feeding impaired the capacity of hepatic ketogenesis during heart failure, another way that can preserve the capacity of ketogenesis should be explored. Alternate-day fasting, which is an alternative to daily caloric restriction, is widely used in experimental and clinical studies, showing obvious health benefits with no significant adverse effects [46–48]. We wondered whether alternate-day KD feeding can be a potential strategy to avoid the adverse effects induced by continuous KD feeding. We found that alternate-day KD feeding is successful in the preservation of the capacity of hepatic ketogenesis and elevation of circulating *β*-OHB in TAC mice. It did not reduce HMGCL content in the liver. As a result, it exerted significant cardioprotective effects against heart failure. Here, we showed that circulating *β*-OHB levels were associated with cardiac oxidative status and cardiac function in TAC mice, suggesting that *β*-OHB plays an important role in mediating the beneficial effects of alternate-day KD feeding. These results suggested that alternate-day KD feeding is a better strategy than continuous KD feeding, which shows milder adverse effects and higher efficiency in ketogenesis during heart failure. These results also reinforced the notion that chronic KD feeding is accompanied with risks for lipid metabolism and ketogenesis disorders, especially in pathological conditions. However, further investigation is required to verify whether alternate-day KD feeding is indeed a potential clinical treatment approach for heart failure.

## 5. Conclusions

Continuous KD feeding failed to protect the heart against heart failure as a result of impairing the capacity of hepatic ketogenesis during heart failure. Mechanistically, continuous KD feeding induced mitochondrial oxidative stress and HMGCL deficiency in the liver, limiting *β*-OHB production in TAC mice. Importantly, we found that alternate-day KD feeding preserved the capacity of hepatic ketogenesis and exerted significant cardioprotective effects against heart failure. These results suggested that alternate-day KD feeding is a better strategy than continuous KD feeding in the treatment of heart failure.

## Data Availability

The data used to support the findings of this study are included within the article.
